# Deficit in Motor Skill Consolidation-Dependent Synaptic Plasticity at Motor Cortex to Dorsolateral Striatum Synapses in a Mouse Model of Huntington’s Disease

**DOI:** 10.1523/ENEURO.0297-19.2020

**Published:** 2020-03-26

**Authors:** Christelle Glangetas, Pedro Espinosa, Camilla Bellone

**Affiliations:** Department of Basic Neuroscience, University of Geneva, 1211 Geneva, Switzerland

**Keywords:** Huntington’s disease, motor skills, synaptic transmission

## Abstract

Huntington’s disease (HD) is a neurodegenerative disease notably characterized by progressive motor symptoms. Although the loss of medium spiny neurons (MSNs) in the striatum has been associated with motor deficits, premanifest patients already present cognitive deficiencies and show early signs of motor disabilities. Here, in a YAC128 HD mouse model, we identified impairment in motor skill consolidation at the age of 11–14 weeks. Using optogenetic stimulation, we found that excitatory synaptic transmission from motor cortex to MSNs located in the dorsolateral part of the striatum (DLS) is altered. Using single pellet reaching task, we observed that while motor skill consolidation is accompanied by a dynamic change in AMPA/NMDA ratio in wild-type (WT) mice, this form of synaptic plasticity does not occur in YAC128 mice. This study not only proposes new meaningful insight in the synaptopathic mechanisms of HD, but also highlights that deficit in motor skill consolidation-dependent synaptic plasticity at motor cortex to DLS synapses represents an early biomarker for HD.

## Abstract (precis)

Huntington’s disease (HD) is a neurodegenerative disease notably characterized by progressive motor symptoms. Although the loss of medium spiny neurons (MSNs) in the striatum has been associated with motor deficits, premanifest patients already present cognitive deficiencies and show early signs of motor disabilities. Here, in a YAC128 HD mouse model, we identified impairment in motor skill consolidation at the age of 11–14 weeks.

## Significance Statement

Huntington’s disease (HD) is a neurodegenerative disease characterized by prominent motor manifestations in addition to nonmotor changes in behavior and cognition. Several studies have provided evidences that the neuropathological hallmark of HD begins and progresses before the conventional diagnosis can be made. Here using an animal model, we identified deficit in motor skill in early stage of the disease. Remarkably these early behavioral deficits are accompanied by aberrant plasticity at synapses between motor cortex and dorsal striatum. This study not only gives a better understanding in the synaptopathic mechanisms of HD, but also highlights that deficit in motor skill consolidation-dependent synaptic plasticity at motor cortex to dorsal striatum synapses represents an early biomarker for HD.

## Introduction

Huntington’s disease (HD) is an autosomal dominant neurodegenerative disease caused by CAG repetition in the gene encoding huntingtin protein (HTT) and is characterized by progressive motor, cognitive and psychiatric symptoms. Neurodegeneration of medium spiny neurons (MSNs) in the striatum is the principal pathologic hallmark of HD (de la Monte et al., 1988). Although MSNs degeneration has been associated with motor deficits, premanifest patients already present cognitive deficiencies (Giralt et al., 2012) and show subtle signs of motor disabilities (Tabrizi et al., 2011). Indeed, slight impairment in motor coordination, in fine motor control of upper extremities and in motor sequence learning have been described in premanifest HD patients (de Boo et al., 1997; Kirkwood et al., 1999, 2000; Tabrizi et al., 2009; Schneider et al., 2010). It has been therefore proposed that fine motor evaluation may represent an early biomarker of the disease (Duff et al., 2008).

Several mechanisms attempted to explain how mutated HTT (mHTT) protein leads to neuronal dysfunction without cell death in premanifest HD. Reduced striatal activity and changes in synaptic properties in the striatum have been described both in human and in mouse models (Milnerwood and Raymond, 2010; Wolf et al., 2012). In particular, increased sensitivity to NMDA (Levine et al., 1999) and increased extrasynaptic NMDAR signaling (Milnerwood et al., 2010) have been observed at early stage of the disease. These data suggest that dysfunctions in NMDA transmission may occur early in the disease progression. NMDARs are heteromeric receptors containing GluN1 subunits together with a combination of GluN2 (A-D) and/or GluN3 (A, B) subunits. Subunit composition determines the receptor’s biophysical and pharmacological properties and changes in NMDAR subunit composition contribute to the pathophysiology of several neurologic diseases (Paoletti et al., 2013). The expression of both GluN2B and GluN3A subunits have been previously linked to HD. Extrasynaptic GluN2B-containing NMDARs are enriched in the striatum of transgenic mice expressing mutated full-length human HD gene (YAC128) at an age preceding motor dysfunctions (Milnerwood et al., 2010). Elevated GluN3A expression has been observed in both HD mouse models and human patients and linked to abnormal excitation of MSNs in the striatum (Marco et al., 2013; Mahfooz et al., 2016). Although early postsynaptic changes in NMDAR-mediated transmission have been described in premanifest HD mouse models, it is still an open question whether these changes only impact survival/death signaling balance and consequent neuronal degeneration or whether they could also be causally link to early behavioral phenotypes.

New motor skill learning is often characterized by a fast-initial phase of improvement of the performance followed by a gradual progress of motor skills. After consolidation, memory becomes long-lasting and can persist for the entire life. Several studies have indicated that the striatal circuits and the synaptic mechanisms engaged during early and late phase of skill learning differ. Specifically, while changes in dorsomedial striatum (DMS) have been predominantly observed during early training, changes in dorsolateral striatum (DLS) have been only detected after extensive training (Yin et al., 2009). Subjects with premanifest HD exhibit learning impairment with no differences in initial performance prior to the time of clinical diagnosis (Shabbott et al., 2013). The neuronal mechanisms underlying these deficits are still largely unknown. Interestingly in mice it has been previously shown that consolidation of motor skills is accompanied by long-lasting changes in glutamatergic transmission onto MSNs and requires striatal NMDAR (Dang et al., 2006; Yin et al., 2009; Lambot et al., 2016). Whether deficits of synaptic plasticity in HD could underlying motor skill deficits represents an interesting hypothesis.

Here, using YAC128 HD mouse model, we found that between the age of 11–14 weeks, mice show impairment in motor skill consolidation. Using optogenetic stimulation, we have observed a decrease in AMPA/NMDA ratio at motor cortex to DLS MSN synapses. This change was accompanied by a change in NMDA receptor subunit composition and by an aberrant NMDA-dependent form of long-term depression (LTD) at motor cortex to DLS when compared with control mice. Remarkably, using single pellet reaching task, we found that motor skill consolidation was accompanied by a reduction in AMPA/NMDA ratio in wild-type (WT) mice. This form of synaptic plasticity was absent in YAC128 mice suggesting that the decreased AMPA/NMDA ratio in YAC128 mice limits consolidation of motor skills in premanifest HD.

## Materials and Methods

### Animals

YAC128 homozygote in a FVB/N background (line 55) crossed into a C57Bl6J background (four back-crosses) were obtained from Perez Otano laboratory (as previously described in Marco et al., 2018). YAC128 homozygous were intercrossed with Tg(Drd1-dtTomato) heterozygous transgenic mice (generous gift from Prof. N. Deglon; C57BL6/j background) to generate YAC128 heterozygous-Drd1-dtTomato heterozygous mice. We then crossed YAC128 heterozygous-Drd1-dtTomato heterozygous mice to generate YAC128 homozygous-Drd1-dtTomato mice (YAC128-D1) selected with real-time quantitative PCR analysis. We then used YAC128 homozygous-Drd1-dtTomato mice from YAC128 homozygous-Drd1-dtTomato mice crossing with YAC128 homozygous or YAC128 homozygous-Drd1-dtTomato mice. Both YAC128 homozygous and YAC128 homozygous Drd1-dtTomato positive mice were used for *in vitro* electrophysiology and behaviors as similar phenotypes were observed regardless of Drd1-tdTomato genotype (data not shown). In parallel, we crossed Tg(Drd1-dtTomato) heterozygous transgenic mice with WT YAC128 mice (selected from YAC128 heterozygous crossing) to generate WT Drd1-dtTomato mice. Both age and genetic background matched WT Drd1-dtTomato transgenic mice without differentiate homozygosity from heterozygosity for Drd1-dtTomato (which can be a limitation point in this study) and C57BL6/j were used as controls in this study. WT and YAC128 mice are not generated from same breeding pairs and parental behaviors were not taken into considerations in this study. Both males and females were respectively housed in groups with food and water *ad libitum* under controlled conditions (22–23°C, humidity 50 ± 5%, 12/12 h light/dark cycle with light on at 7:00 A.M.). All the procedures performed at the UNIL and UNIGE compiled with the Swiss National Institutional Guidelines on Animal experimentation and were approved by the Swiss Cantonal Veterinary Office Committee for Animal Experimentation. Vd 3016.d license authorization.

### Electrophysiology

We prepared 250-μm-thick coronal slices containing DLS following the experimental injection protocols described in the text. Slices were kept in artificial cerebrospinal fluid containing 119 mM NaCl, 2.5 mM KCl, 1.3 mM MgCl_2_, 2.5 mM CaCl_2_, 1.0 mM NaH_2_PO_4_, 26.2 mM NaHCO_3_, and 11 mM glucose, bubbled with 95% O_2_ and 5% CO_2_. Slices were maintained 30 min in bath at 30°C and then at room temperature. Whole-cell voltage-clamp recording techniques were used (37°C, 2–3 ml min^−1^, submerged slices) to measure the holding currents and synaptic responses of DLS MSN. The internal solution contained 130 mM CsCl, 4 mM NaCl, 2 mM MgCl_2_, 1.1 mM EGTA, 5 mM HEPES, 2 mM Na2ATP, 5 mM sodium creatine phosphate, 0.6 mM Na3GTP, and 0.1 mM spermine. Currents were amplified, filtered at 5 kHz and digitized at 20 kHz.

Access resistance was monitored by a hyperpolarizing step of −4 mV at each sweep, every 10 s. The cells were recorded at the access resistance from 10 to 25 MΩ for MSN. Data were excluded when the resistance changed >25%. Synaptic currents were evoked by intrastriatal electrical stimulation at 0.1 Hz and 0.05–0.1 ms of duration. For optogenetic experiments, we stimulated the glutamatergic fibers from motor cortex or from the thalamus in the DLS. The stimulus was delivered at 0.1 Hz and the duration was 1–3 ms. The experiments were conducted in the presence of GABAA receptor antagonist picrotoxin (100 μM); the AMPAR-EPSCs were pharmacologically isolated by application of the NMDAR antagonist D-APV (50 μM) and NMDAR EPSCs were recorded at +40 mV in presence of the AMPAR blocker NBQX (10 μM). Representative example traces are shown as the average of 15–20 consecutive EPSCs typically obtained at each potential. The rectification index of AMPARs is the ratio of the chord conductance calculated at negative potential (−60 mV) divided by the chord conductance at positive potential (+40 mV). The analysis of the decay time of NMDAR-mediated EPSC was conducted as described previously and the Ifenprodil sensitivity was calculated as the percentage of NMDAR-EPSC amplitude reduction (at +40 mV) after 20–25 min of continuous Ifenprodil (3 μM, GluN2B-containing NMDAR antagonist) bath-application compared with baseline. The time interval between the two stimulations for the paired pulse ratio (PPR) measurement was 50, 100, and 300 ms (interstimulation interval, ISI) and the ratio was obtained by dividing the EPSC2 by EPSC1 amplitude at –60 mV.

For the in vitro validation of the optogenetic experiment and the strontium chloride experiment, the internal solution contained 140 mM K-gluconate, 2 mM MgCl_2_, 5 mM KCl, 0.2 mM EGTA, 10 mM HEPES, 4 mM Na_2_ATP, 0.3 mM Na3GTP, and 10 mM creatine-phosphate. Blue-light was delivered through the 40× objective focused on the cell soma. The Synaptic responses were collected with a Multiclamp 700B-amplifier (Axon Instruments), filtered at 2.2 kHz, digitized at 10 Hz, and analyzed online using Igor Pro 6 software (Wavemetrics).

I-V curves of pharmacologically isolated NMDARs were generated holding the cells at different membrane potential for 5 min each and normalizing EPSCs at 40 mV.

Asynchronous evoked EPSC (aEPSC). For this experiment, 3 mM of SrCl_2_ was added in the aCSF solution instead of the CaCl_2_. The internal solution contained: 140 mM K-gluconate, 2 mM MgCl_2_, 5 mM KCl, 0.2 mM EGTA, 10 mM HEPES, 4 mM Na_2_ATP, 0.3 mM Na_3_GTP, and 10 mM creatine-phosphate. Cells were hold at –70 mV, and picrotoxin was added in this external bath. Asynchronous events were measured during 180-ms period, between 20 and 200 ms after stimulation (Choi and Lovinger 1997). Quantal events were detected and analyzed using Mini analysis program version 6.0.

The plasticity experiments were recorded at −40-mV holding potential, we measured 10 min of baseline response at 0.1 Hz, followed by 5 min of stimulation at 1 Hz. Then we recorded the EPSCs for 40 min.

### Stereotaxic injections

AAV5-CamKII-hChR2(H134R)-EGFP virus has been injected in the motor cortex in four- to seven-week-old mice. Anesthesia was induced and maintained with a mixture of oxygen and isoflurane. The animals were then placed on the stereotaxic frame (Stoelting Co) and a single or bilateral craniotomy was made over motor cortex at following stereotaxic coordinates: M1: AP +1.18 mm, ML 1.21 mm, DV 0.65 mm from bregma; M2: AP +1.18 mm, ML 0.60 mm, DV 0.50 mm from bregma and for the thalamus at following coordinates: AP −2.30 mm, ML 0.6 mm, DV 3 mm from bregma. The virus was injected with graduated pipettes (Drummond Scientific Company) at the rate of 100 nl/min for a total volume of 200 nl per injection side. For all the experiments the virus was incubated for at least four weeks, when expression was clearly identifiable by the reporter protein expression, before proceeding with further manipulations.

### Circular corridor test

Mice were placed in a circular corridor (30 cm in diameter) and were allowed to freely explore the circular corridor for a 30-min period in 11-lux illumination condition. Total distance traveled and velocity during the session were automatically recorded (Ethovision, Noldus). Arena was cleaned with 1% acetic acid and dried between each test.

### Open field test

Mice were placed in a square open field (42 × 42 cm) and were allowed to freely explore the open field for a 10-min period in 11-lux illumination condition. Total distance traveled and velocity during the session were automatically reported (Ethovision, Noldus). The arena was cleaned with 1% acetic acid and dried between each test.

### Single pellet reaching task test

**Figure 2.**
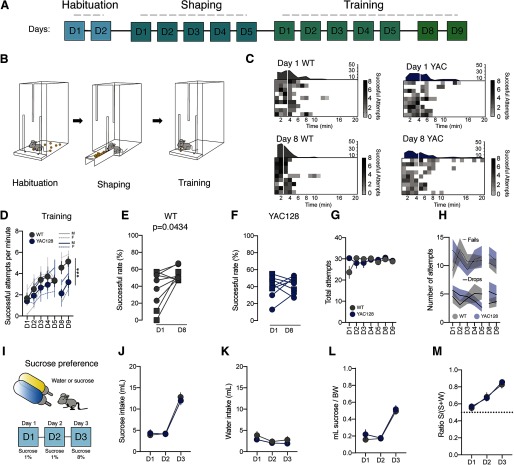
Forelimb motor skill consolidation is impaired in premanifest stage in YAC128 mice. ***A***, ***B***, Timeline (***A***) and experimental schematic (***B***) of single pellet reaching task. ***C***, Heat map of successful attempts in WT and YAC128 mice at day 1 and day 8. Each horizontal line in the heat map represents the performance of an individual mouse. Each vertical white line represents the time to reach 50% of the pellets. ***D***, Average speed of success over the training phase of single pellet reaching task in WT and YAC128 mice. ***E***, ***F***, Scatter plot and group mean of the success rate at day 1 versus day 8 of the single pellet reaching task within WT (***E***) and YAC128 mice (***F***). ***G***, Kinetic of total attempts (including success, drop and failed attempts) across single pellet reaching task training days in WT and YAC128 mice. ***H***, Kinetic of fail rate and drop rate during single pellet training in WT and YAC128 mice. ***I***, Experimental schematic. ***J***, Group mean sucrose consumption across days. ***K***, Group mean water consumption across days. ***L***, Group mean sucrose consumption normalized to bodyweight across days. ***M***, Sucrose preference at day 1, day 2, and day 3 in WT and YAC128 mice. Square symbols represent female mice and circles represent males. Error bars show SEM; ***p < 0.001.

To evaluate motor skill learning, a single pellet reaching task was performed. This paradigm requires a precise and coordinated sequence of movements of the forelimb in a serial order. Mice are trained to extend their forelimbs through a narrow slit to grasp and retrieve millet pellets (food) positioned at a fixed location as described in Chen et al., 2014. First, mice are placed on a food restriction schedule, 90% of their free feeding body weight (Chen et al., 2014; Lambot et al., 2016). In detail, food restriction starts 2 d prior experiment to initiate bodyweight loss. In a second step, group and individual habituation have been done in the training chamber. In details, two cage mate mice are placed in the training chamber (custom made transparent Plexiglas training chamber 20 cm tall, 15 cm deep, and 8.5 cm wide that contains three vertical slits) at the same time with 20 millet pellets inside the chamber for 20 min. The next day, a single habituation as previously described has been accessed (Fig. 2*A*,*B*). Then, to determine the forelimb dominance, a food platform with millet pellets was placed in front of the training chamber to allow the accessibility of the pellets to the mouse through vertical slit of the training chamber. This shaping phase is achieved when two criteria were encountered (1) the mouse conducts 20 reaching attempts within 20 min and (2) >70% reaching attempts are performed with one forelimb. If the mouse does no attempt these criteria within one week, the mouse was then excluded from the experiment. After this shaping phase (5 d), a single-pellet training started (8 d). During the training phase, mice are trained to reach single pellet for 20 min/d. After training, mice returned to their home cage and were kept under food restriction. Three responses were manually scored: success (grasp the pellet with the preferred paw and put it in the mouth), drop (grasp the pellet with the preferred paw and release it before to put in the mouth) and fail (ca not grasp the pellet with the preferred paw). Speed of success is defined as the number of successful reaches per minute. Success rate is the number of successful reaches divided by total reaching attempts [success/(success + drop + fail attempts)] expressed in percentage. Fail rate is the number of fail reaches divided by total reaching attempts [fail/(success + drop + fail attempts)] expressed in percentage. During the entire experiment, mice are group-housed (age and sex matches). All the procedure was recorded with a camera JVC model no. GZ-R430BE. 1 YAC128 and 1 WT mice have been excluded according to the exclusion criteria.

### Rotarod test

Mice were brought 30 min prior the experiment in the room to allow acclimatization. The rotarod apparatus (Ugo Basile, Biological Research Apparatus) consisted of a plastic roller with small grooves running along its turning axis. Mice received two trials per day for four consecutive days, then the training was interrupted for 2 d, after this period the training restarted for two additional days. The protocol consists in a classical accelerated rotarod (Southwell et al., 2009) from five rotations per minute (RPM) to 40 RPM within 240 s ramping over a maximum duration of 300 s with 10-min interval session break. We scored the mouse fall latency in seconds of each last trial session per day. Mice that did not fall during experiment were scored as 300 s.

### Swimming tank test

**Figure 1.**
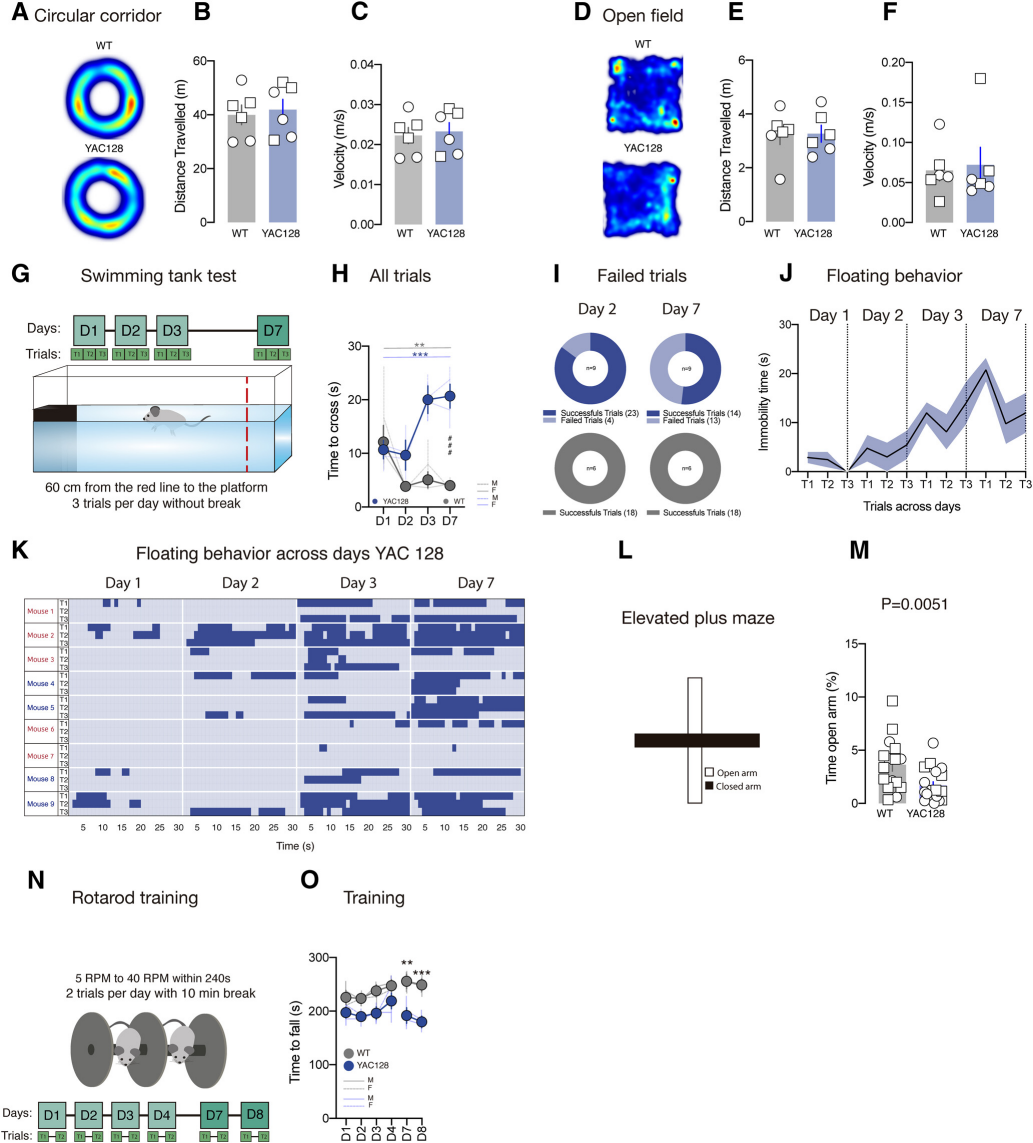
Motor consolidation alterations in swimming tank test and rotarod task in premanifest stage in YAC128 mice. ***A***, ***D***, Activity trail plots. ***B***, ***E***, Group mean of the distance traveled in circular corridor (***B***) or in open field (***E***) in WT and YAC128 mice. ***C***, ***F***, Group mean of the velocity in circular corridor (***C***) or in open field (***F***) in WT and YAC128 mice. ***G***, Experimental schematic. ***H***, Time course of cross latency in swimming tank test in WT and YAC128 mice. ***I***, Quantification of failed trials at day 2 and day 7 of swimming tank test in WT and YAC128 mice. ***J***, Quantification of floating behavior across training in YAC128 mice. ***K***, Floating behavior across swimming tank test training in YAC128 mice. Each horizontal line represents the floating behavior of an individual mouse during the 30 s trial for the trial 1 (t1), the trial 2 (t2), and the trial 3 (t3) at day 1, day 2, day 3, and day 7. Each episode of floating behavior is represented in dark blue. Females are represented in blue color while male mice are labeled in red color. ***L***, Experimental schematic. ***M***, Group mean of the time in open arms expressed in percentage in the elevated plus maze WT and YAC128 mice. ***N***, Experimental schematic. ***O***, Time course of the fall latency in rotarod in WT and YAC128 mice. Square symbols represent female mice and circles represent males. Error bars show SEM M.: male, F.: female; *p < 0.05, **p < 0.01, ***p < 0.001; ###*p* < 0.001 YAC D7 versus WT D7.

To measure swimming behavior, we used a swimming tank apparatus build of Plexiglas, the dimensions were 100 cm long, 30 cm high, and 6 cm wide with an escape platform in one extremity (6 × 6 cm and 20 cm high; Carter et al., 1999). The tank was filled with water (26–27°C) until the escape platform protrudes 1–2 cm above the water level. In the opposite side of the platform, a vertical red line indicates the starting point located at 60 cm from the platform. The first day of training, the animals were deposited in the tank and when necessary were conducted to reach the platform. From day 2 of training, animals were deposited and slightly conducted until the red line. The task consists in three consecutive trials (∼10 s between trails), performed daily from day 1 to 3. After 3 d of recovery, we run the last training session (day 7). We measured the time to swim the 60 cm of distance from the red line to the platform. Trials were finished when mice reached and climbed on the platform. Given that YAC128 mice expressed a floating behavior during this task we set a threshold time of 30 s, if mice completed the task in >30 s, the trials were counted as failed. In Figure 1*H*, we used Drd1 td tomato mice as WT and Drd1td tomato-YAC128 as YAC128. We have also performed the same experiments in C57BL6/J and YAC128 and there was no difference in the performance with insertion of D1 td tomato allele (data not shown).

### Elevated plus maze

The elevated plus maze consisted in a platform of four opposite arms (40 cm) two of them are open and two are closed arms (enclosed by 15 cm high walls). The apparatus was elevated at 55 cm from the floor. The task was recorded and analyzed with the software Ethovision (Noldus) and we measured the time spent in each arm in trials of 5 min. The luminosity of the room was 11–12 lux in the open arms.

### Sucrose preference test

Mice were housed individually after the end of the single pellet reaching task for the duration of this task (3 d) and had access to standard lab chow and tap water throughout the experiment. At 6:00 P.M., they were exposed to two drinking bottles, one containing water and the other one with a sucrose solution. During the first 2 d, sucrose was given at 1% and the third day, the sucrose solution was given at 8% concentration. Every day, the sucrose and the water consumption were weighted. Water and sucrose bottle positions were counterbalanced to avoid any confounding effect of side preference. Sucrose and water consumption were measured for each mouse and a sucrose preference ratio was calculated [sucrose consumed/(sucrose consumed + water consumed)]. One WT mouse has been excluded for the day 1 of sucrose consumption due to a problem in the sucrose bottle.

### Real-time PCR

Real-time PCR was performed by microsynth company to determine the genotype of the transgenic YAC128 mice. Genomic DNA was isolated from ear punch or postmortem tail biopsies and analyzed by real-time PCR specific for huntingtin gene and β-actin. The resulting Ct values are used for relative quantification of the copy-number of human specific huntingtin (HD) in the provided samples according to the following equation: ΔCt = Ct (β actin) − Ct (HD). The gene expression fold change, normalized to the β-actin and relative to the control sample, was calculated as 2 ΔCt. Values close to 1 corresponds to HD homozygous while values lower than 0.5 corresponds to HD heterozygous mice. All samples were run in triplicate. The following primers used for the real-time PCR reaction were:

### HD primer

F 5′-GAAAGTCAGTCCGGGTAGAACTTC-3′

R 5′-CAGATACCCGCTCCATAGCAA-3′

### Mouse b-actin primers

F 5′-ACGGCCAGGTCATCACTATTG-3′

R 5′-CAAGAAGGAAGGCTGGAAAAGA-3′

F refers to forward primer and R refers to reverse primer.

Briefly, real-time PCR was assayed in a total volume of 20 μl reaction mixture containing 2.5 μl of diluted cDNA, 10 μl SYBR Green PCR master mix (Applied Biosystem), 1 μl primer F (5 pmol/μl), 1 μl primer R (5 pmol/μl), 5.5 μl H_2_O. PCR thermal conditions were done with a step at 50°C for 2 min, a 10 min at 95°C, followed by 40 cycles of denaturation for 15 s at 95°C and annealing/primer elongation for 1 min at 60°C.

### Drug and viruses

AAV5-CamKII-hChR2(H134R)-EYFP virus (2.8 × 10e^12^ viral molecules/ml, UNC GTC Vector core), D-AP5 (0106, Tocris), Picrotoxin (1128, Tocris), NBQX (0373, Tocris), and Ifenprodil hemitartrate (0545, Tocris).

### Statistical analysis

Normality was checked with the Shapiro-Wilk criterion and when violated, non-parametric statistics were applied (Mann–Whitney and Kruskal–Wallis). When samples were normally distributed, data were analyzed with independent or paired two-tailed samples *t* tests, one-way, two-way, or repeated measures ANOVA followed if significant by *post hoc* tests. All error bars represent the mean ± SEM, and the significance was set at *p* < 0.05. Data were analyzed using the GraphPad Prism 5 and 7, and graphs were created using the GraphPad Prism 5 and 7. Outliers were defined as higher than mean ± 2 SD. In all the electrophysiological experiments we excluded 10 cells in total, six WT and four YAC128.

## Results

YAC128 mice show strong motor dysfunctions when they reached 10 months (Marco et al., 2013) while earlier detection of motor disturbance in this model is more controversial (Slow et al., 2003; Van Raamsdonk et al., 2005, 2007; Pouladi et al., 2009; Southwell et al., 2009). To verify whether subtle deficits in motor behavior could be observed at earlier time points, we tested mice between 11 and 14 weeks. We did not detect any gross impairment in locomotor activity as indicated by the absence of differences in traveled distance and velocity between WT and YAC128 mice in the circular corridor (distance traveled: WT: 39.98 ± 3.64 m, *N* = 6 mice; YAC128: 41.91 ± 3.91 m, *N* = 6 mice, *t*_(10)_ = 0.3603 unpaired *t* test, *p* > 0.05; velocity WT: 0.02 ± 0.002 m/s; YAC128: 0.02 ± 0.002 m/s, *t*_(10)_ = 0.3419 unpaired *t* test, *p* > 0.05**;** Fig. 1*A–C*) and in the open field tests (distance traveled: WT: 3.24 ± 0.37 m; YAC128: 3.27 ± 0.31 m, *t*_(10)_ = 0.06,773 unpaired *t* test, *p* > 0.05; velocity WT: 0.07 ± 0.01 m/s; YAC128: 0.07 ± 0.02 m/s, *U* = 15 Mann–Whitney test, *p* > 0.05; Fig. 1*D–F*). However, in the swimming tank test (Fig. 1*G*) while independently on the genotype, mice present equivalent initial behavior at day 1 and improve the time to cross the tank at day 2, only WT mice maintain their performance over the following days (Friedman test for WT, *F*_(4)_ = 11.40 *p* = 0.004; *N* = 6 mice, Friedman test for YAC128 mice, *F*_(4)_ = 18.21 *p* = 0.0004, *N* = 9 mice, Mann–Whitney test for WT D1 vs YAC128 D1 *p* = 0.9305, Mann–Whitney test for WT D7 vs YAC128 D7 *p* = 0.0004; Fig. 1*H*). During the test, in YAC128 mice we also observed an increased number of failed trials (Fig. 1*I*) compared with controls and the appearance of floating behavior (Fig. 1*J*,*K*). Although we cannot exclude that YAC128 mice took more time to reach the platform across days compared with WT as consequence of the emergence of floating behavior, our data suggest that early stages of the disease are characterized by deficits in consolidation of the motor performance in the swimming tank test.

We performed an elevated plus maze to evaluate whether YAC128 mice present an anxiety phenotype at this early stage. We observed that YAC128 mice spent less time in the open arms compared with WT (WT 3.650 ± 0.6%, *N* = 16 mice, YAC128 1.686 ± 0.3%, *N* = 19 mice, *p* < 0.005; Mann–Whitney test; Fig. 1*L*,*M*), suggesting an anxiety-like phenotype in these mice at early stage of the disease.

To assess motor learning abilities of YAC128 mice, we used the accelerated rotarod task (Fig. 1*N*). Rotarod task allow us to observe the acquisition and the consolidation of a new motor skill (Karni et al., 1998). While no differences in the time to fall were observed at days 1 and 4 (Fig. 1*O*), YAC128 mice did not improved their performance over the days and differences between mice were observed at day 7 and day 8 (two-way ANOVA main effect of genotype *F*_(1,96)_ = 13.85, *p* < 0. 001 followed by Bonferroni *post hoc*, WT D7 255.6 ± 13.15 s, *N* = 20 mice, YAC128 D7 191.9 ± 14.72 s, *N* = 14 mice, *p* < 0.005; Fig. 1*O*).

To study fine motor skill learning involving forelimb dexterity, we then adopted the single-pellet reaching task. In this task, food restricted mice were trained to extend their forelimbs through a narrow slit to grasp and to retrieve food pellets positioned in a fixed location. After 2 d of habituation, mice underwent 5 d of shaping followed by a period of training (Fig. 2*A*,*B*; see Materials and Methods). YAC128 and control mice showed similar performance at the first training session. Strikingly, while control mice still improved the successful attempts per minute after a break (day 8), YAC128 mice did not enhance their performance (Fig. 2*C*,*D*; two-way ANOVA interaction effect *F*_(2,48)_ = 4.32, *p* < 0.05, followed by a Bonferroni *post hoc* WT D8 4.578 ± 0.5809 successful attempts per minute *N* = 9 mice, YAC128 D8 2.133 ± 0.4052 successful attempts per minute, *N* = 9 mice *p* < 0.01; Fig. 2*D*). In addition, successful rate increased between day 1 versus day 8 in WT while it remained unchanged in YAC128 mice (paired *t* test for WT D1: 37.56 ± 7.120, D8: 53.89 ± 2.6, *t*_(8)_ = 2.397, *p* < 0.05 and for YAC128 D1: 39.44 ± 4.1 D8: 43.0 ± 3.0, *t*_(8)_ = 0.7702, *p* > 0.4633; Fig. 2*E*,*F*). Alteration in single pellet reaching task in YAC128 mice does not account for difference in bodyweight compared with WT across the task (data not shown), neither for differences in total attempts or fail rate across days between groups (fails: two-way ANOVA interaction effect *F*_(6,112)_ = 0.714, *p* > 0.05, no main effect; drop: two-way ANOVA interaction effect *F*_(6,112)_ = 1.071, *p* > 0.05, and no main effect; Fig. 2*G*,*H*), nor for general anhedonia (lack of interest in a rewarding stimulus) as indicated by no difference in sucrose preference test (sucrose preference, WT D2: 0.67 ± 0.06; YAC128 D2: 0.66 ± 0.03; Mann–Whitney, *p* > 0.05; WT D3: 0.82 ± 0.05; YAC128 D3: 0.85 ± 0.01, Mann–Whitney, *p* > 0.05; Fig. 2*I–M*). These data suggest that at early stages of the disease, HD mice present difficulties in the consolidation of newly acquired motor skills.

**Figure 3.**
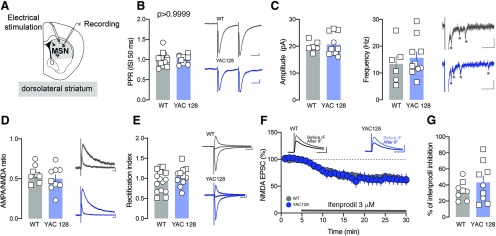
No major differences on synaptic strength with electrical stimulation in YAC128 MSN. ***A***, Experimental schematic. ***B***, Group mean PPR for WT and YAC128 MSN. Right, Example traces of AMPAR-EPSC at −60 mV in WT and YAC128 MSN. Scale bar: 20 ms, 50 pA. ***C***, Group mean amplitude and frequency of asynchronous evoked events in WT and YAC128 MSN. Right, Example traces of evoked AMPAR-aEPSCs recorded at −70 mV. Star indicating an asynchronous event detected. Scale bar: 50 ms, 25 pA. ***D***, Group mean AMPA/NMDAR ratio calculated in WT and YAC128 MSN. Right, Example traces of evoked AMPAR- and NMDAR-EPSCs at +40 mV. Scale bar: 20 ms, 50 pA. ***E***, Group mean RI calculated in WT and YAC128 MSN. Right, Example traces of evoked AMPAR-EPSCs recorded at −60, 0, and + 40 mV. Scale bar: 20 ms, 50 pA. ***F***, Time course of NMDAR-EPSC amplitude during ifenprodil application for WT and YAC128 MSN and associated example traces in WT and YAC128 mice in the inset. ***G***, Group mean ifenprodil inhibition calculated in WT and YAC128 MSN. Scale bar: 50 ms, 50 pA. Square symbols represent female mice and circles represent males.

Changes in excitatory synaptic transmission in the striatum have been previously described in symptomatic HD mouse models. Here, we first investigated whether early behavioral traits were accompanied by specific changes in glutamatergic synaptic transmission onto MSN in the DLS. Using intra-striatal electrical stimulation (Fig. 3*A*), we did not detect changes in presynaptic release properties (WT 1.01 ± 0.04, *n* = 14 neurons; YAC128 1.01 ± 0.03, *n* = 10 neurons, unpaired *t* test, *t*_(22)_ = 0, *p* > 0.05, 1 outlier WT; Fig. 3*B*), neither in the amplitude and frequency of strontium-evoked asynchronous AMPAR events (WT 20 ± 0.81 pA, *n* = 6 neurons; YAC128 20.62 ± 1.29 pA, *n* = 10 neurons, Mann–Whitney test, *U* = 27 *p* > 0.05; WT 13.39 ± 3 Hz; YAC128 15.69 ± 2.21 Hz unpaired *t* test, *t*_(14)_ = 0.6266 *p* > 0.05; Fig. 3*C*). Furthermore, we did not detect change in the strength of synaptic transmission measured as AMPA/NMDA ratio (WT 0.56 ± 0.03, *n* = 9 neurons; YAC128 0.50 ± 0.06, *n* = 9 neurons unpaired *t* test, *t*_(15)_ = 1.054 *p* > 0.05; Fig. 3*D*). When we pharmacologically isolated AMPARs we did not detect any change in rectification index (WT 0.93 ± 0.07, *n* = 14 neurons; YAC128 1.05 ± 0.07, *n* = 10 neurons, unpaired *t* test, *t*_(24)_ = 1.165 *p* > 0.05; Fig. 3*E*), suggesting that YAC128 mice at this age do not present GluA2-lacking AMPARs. When we pharmacologically isolated NMDARs, ifenprodil sensitivity was not different between YAC128 and control mice (WT 33.09 ± 4.13%, *n* = 8 neurons; YAC128 43.13 ± 8.18%, *n* = 8 neurons, unpaired *t* test, *t*_(16)_ = 0.9795 *p* > 0.05; Fig. 3*F*,*G*), suggesting that NMDA subunit composition was not changed in YAC128 mice compared with control.

**Figure 4.**
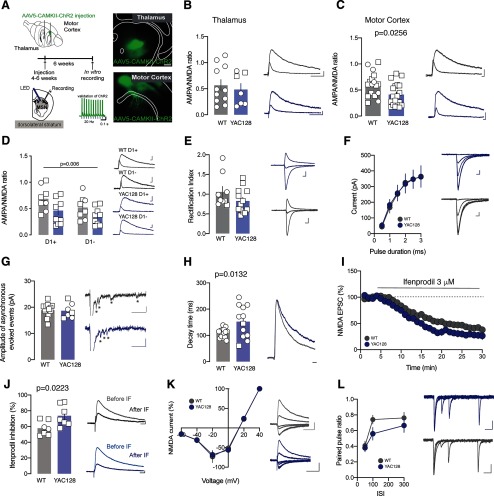
NMDAR transmission dysfunction at motor cortex to DLS MSN in YAC128 mice. ***A***, left, Experimental schematic. Left down, *In vitro* validation of 20-Hz blue light stimulation protocol. Scale bar: 0.1 s, 10 mV. Right, Epifluorescent image of AAV5-CamKII-hChR2(H134R)-EGFP injection in the thalamus (top) or in the motor cortex (down). ***B***, ***C***, Group mean AMPA/NMDAR ratio calculated in WT and YAC128 MSN at thalamo-dorsolateral synapses (***B***) or at motor cortex to dorsolateral synapses (***C***). Right, Example traces of evoked AMPAR- and NMDAR-EPSCs at +40 mV. Scale bar: 50 ms, 25 pA (***B***) and 10 ms, 50 pA (***C***). ***D***, Group mean AMPA/NMDAR ratio calculated in WT-D1^+^ MSN, WT-D1^–^ MSN, YAC128-D1^+^ MSN and YAC128-D1^–^ MSN at motor cortex to dorsolateral synapses. Scale bar: 10 ms, 50 pA. ***E***, Group mean RI calculated at motor cortex to DLS synapses in WT and YAC128 MSN. Right, Example traces of evoked AMPAR-EPSCs recorded at −60, 0, and +40 mV. Scale bar: 10 ms, 50 pA (for YAC128) and 10 ms, 50 pA (for WT). ***F***, I-O relationship of motor cortex glutamatergic transmission established by the stimulation duration (synaptic input) and the amplitude of the EPSC (output) in slices from WT and YAC128 MSN. Right, Representative EPSCs evoked by motor cortex terminal stimulation in DLS recorded at −60 mV in WT and YAC128 MSN. Scale bar: 10 ms, 50 pA. ***G***, Group mean amplitude of asynchronous evoked events in WT and YAC128 MSN. Right, Example traces of evoked AMPAR-aEPSCs recorded at –70 mV. Star indicating an asynchronous event detected. Scale bar: 50 ms, 25 pA. ***H***, Group mean decay time of NMDAR–EPSCs at +40 mV in WT and YAC128 MSN. Right, Example traces of NMDAR-EPSC at +40 mV. ***I***, Time course of NMDAR-EPSC amplitude during ifenprodil application for WT and YAC128 MSN. Scale bar: 15 ms. ***J***, Group mean ifenprodil inhibition calculated in WT and YAC128 MSN. Right, Example traces of NMDAR-EPSCs during ifenprodil (3 μM) bath application. Scale bar: 20 ms, 25 pA. ***K***, I-V plots of normalized and averaged NMDAR-EPSCs of motor cortex to DLS MSN in WT and YAC128 mice and their associated example traces. Scale bar: 50 ms, 50 pA. ***L***, Group mean PPR recorded at interval of 50, 100, and 300 ms for WT and YAC128 MSN evoked by motor cortex stimulation. Right, Example traces of AMPAR-EPSC at −60 mV in WT and YAC128 MSN. Scale bar: 50 ms, 25 pA. Square symbols represent female mice and circles represent males.

DLS receives major glutamatergic inputs from motor cortex and thalamus. These inputs have different functional properties and early deficits within specific circuits may lead to deficits in motor skill learning. Using optogenetic tools, we here investigated whether we could detect changes in glutamatergic transmission from motor cortex and thalamus onto DLS MSNs (Smith et al., 2004; Wall et al., 2013; Guo et al., 2015). First, we injected channelrodhopsin (ChR2)-expressing virus in the motor cortex and in the thalamus in control and YAC128 mice (Fig. 4*A*). After six weeks, acute brain slices were obtained and recording were performed from MSN of the DLS. We did not detect differences in the strength of transmission at thalamo-DLS synapses (WT 0.57 ± 0.11, *n* = 11 neurons; YAC128 0.48 ± 0.11, *n* = 7 neurons unpaired *t* test, *t*_(16)_ = 0.4942 *p* > 0.05; Fig. 4*B*). By contrast, when we recorded light-evoked synaptic transmission from motor cortex inputs to DLS MSNs, we found a decreased AMPA/NMDA ratio in YAC128 compared with WT (WT 0.57 ± 0.04, *n* = 22 neurons; YAC128 0.41 ± 0.05, *n* = 19 neurons Mann–Whitney, *U* = 124, *p* < 0.05; Fig. 4*C*) affecting both D1^+^ MSN and D1^–^ MSNs (WT D1^+^ 0.642 ± 0.06, *n* = 8 neurons, WT D1^–^ 0.554 ± 0.08, *n* = 7 neurons, YAC128-D1^+^ 0.426 ± 0.08, *n* = 9 neurons, YAC128-D1^–^ 0.355 ± 0.05, *n* = 9 neurons, two-way ANOVA interaction effect *F*_(1,31)_ = 0.4294, *p* > 0.05, Genotype main effect *F*_(1,31)_ = 8.518, *p* = 0.006; Fig. 4*D*). The rectification index of AMPAR-mediated transmission (WT 0.95 ± 0.1, *n* = 8 neurons; YAC128 0.83 ± 0.06, *n* = 13 neurons Mann–Whitney test, *U* = 38 *p* > 0.05; Fig. 4*E*), amplitude of the optically-induced AMPAR mediated currents (WT, *n* = 8 neurons; YAC128, *n* = 13 neurons; Fig. 4*F*) as well as the amplitude of strontium-evoked asynchronous AMPAR events (WT 18.86 ± 0.57 pA, *n* = 16 neurons; YAC128 18.66 ± 1.07 pA, *n* = 7 neurons unpaired *t* test, *t*_(21)_ = 0.1854 *p* > 0.05; Fig. 4*G*) did not differ between control and YAC128 mice. Furthermore, we did not observe changes in the PPR measured at different time intervals (WT 50 ms: 0.41 ± 0.06, *n* = 15 neurons; YAC128 50 ms: 0.38 ± 0.05, *n* = 11 neurons unpaired *t* test, *t* = 0.3778 *p* > 0.05, WT 100 ms: 0.74 ± 0.06; YAC128 100 ms: 0.56 ± 0.08 unpaired *t* test, *t* = 1.938, *p* = 0.064, WT 300 ms: 0.76 ± 0.08; YAC128 300 ms: 0.61 ± 0.08 unpaired *t* test, *t* = 0.8576 *p* > 0.05; Fig. 4*L*). These data indicate that the decrease in AMPA/NMDA ratio specifically observed at motor cortex to DLS synapses was not accompanied by major changes in AMPAR-mediated transmission and suggest therefore that the reduction of the ratio may be the consequence of an increase in NMDA-mediated current.

To better describe possible changes in NMDAR-mediated current at motor cortex to DLS synapses, we pharmacologically isolated optogenetically-induced NMDAR currents and characterized the NMDAR subunit composition. NMDA-EPSC recorded from YAC128 mice presented a slower decay time (WT 110 ± 4.1 ms, *n* = 16 neurons; YAC128 152.5 ± 17.72 ms, *n* = 12 neurons unpaired *t* test, *t*_(26)_ = 2.662, *p* < 0.05; Fig. 4*H*) and an increased ifenprodil sensitivity (WT 57.76 ± 3.42%, *n* = 7 neurons; YAC128 73.67 ± 5.01%, *n* = 7 neurons unpaired *t* test, *t*_(12)_ = 2.621 *p* < 0.05; Fig. 4*I*,*J*). Furthermore, we could not find changes in current/voltage relationship compared with control mice (WT *n* = 7 neurons, YAC128 *n* = 7 neurons; Fig. 4*K*). These data indicated an enriched GluN2B subunit composition at motor cortex to DLS synapses at early stages of HD diseases but no changes in GluN3A contents.

**Figure 5.**
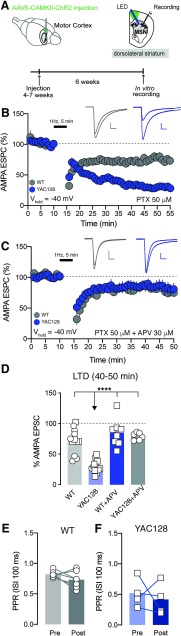
Aberrant NMDAR dependent LTD at motor cortex to DLS MSN in YAC128 mice. ***A***, Experimental schematic. ***B***, ***C***, Kinetic of AMPA EPSC amplitude normalized to baseline at motor cortex to DLS MSN after low-frequency stimulation (1 Hz, 5 min) in WT and YAC128 groups with picrotoxin (50 μM; ***B***) or with picrotoxin and APV (30 μM; ***C***). Top, Example traces pre and post 1 Hz, 5 min. ***D***, Quantification of AMPA EPSC amplitude normalized to baseline at motor cortex to DLS MSN after low-frequency stimulation in WT and YAC128 groups without and with APV application. ***E***, ***F***, PPR pre and post low-frequency stimulation protocol at motor cortex to DLS MSN in WT (***E***) and YAC128 mice (***F***). Scales bar: 20 ms, 50 pA. Square symbols represent female mice and circles represent males. Error bars show SEM; ****p < 0.0001.

NMDAR subunit composition is crucial for the induction of LTD in the DLS (Brigman et al., 2010). We predicted that changes in NMDAR current would therefore impact the induction of this form of synaptic plasticity. We observed that 1-Hz stimulation for 5 min induced a significantly stronger NMDA-dependent LTD at motor cortex to DLS synapses in YAC128 compared with control mice (two-way ANOVA interaction effect *F*_(1,35)_ = 10.45, *p* = 0.0027 followed by a Bonferroni *post hoc* WT: 28.21 ± 5.84% of depression, *n* = 12 neurons vs YAC128 69.31 ± 2.81% of depression, *n* = 13 neurons, *p* < 0.0001; Fig. 5*A*,*B*,*D*). However, when we blocked the NMDA receptor with APV, we did not observe changes between genotypes in the magnitude of the LTD (YAC128 69.31 ± 2.81% of depression vs YAC128 + APV 20.54 ± 2.48% (*n* = 6 neurons) of depression, *p* < 0.0001; Fig. 5*C*,*D*). In addition, this stimulation protocol did not elicit changes in PPR in both groups (WT pre: 0.82 ± 0.02; WT post: 0.74 ± 0.05, *n* = 7 neurons paired *t* test, *t* = 1.593, *p* > 0.05; YAC128 pre: 0.52 ± 0.12; YAC128 post: 0.42 ± 0.13, *n* = 4 neurons paired *t* test, *t* = 0.5259, *p* > 0.05; Fig. 5*E*,*F*).

Altogether these data indicate that at early stage of HD disease there are specific changes in NMDA-mediated currents at motor cortex to DLS synapses and that these changes prime the synapses for changes in NMDA-dependent form of synaptic plasticity.

**Figure 6.**
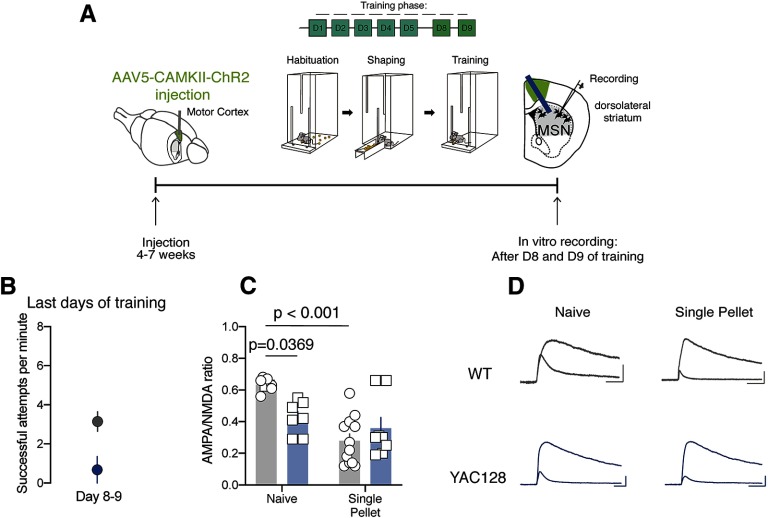
Motor training induced motor cortex to DLS MSN plasticity is occluded in YAC128 mice. ***A***, Experimental schematic. ***B***, Average speed of success at the end of the training phase of single pellet reaching task (day 8 to day 9) in WT and YAC128 mice. ***C***, ***D***, Group mean AMPA/NMDAR ratio calculated in WT and YAC128 MSN in naive group or after single pellet reaching task training. Right, Example traces of evoked AMPAR- and NMDAR-EPSCs at +40 mV. Scales bar: 50 ms, 100 pA. Error bars show SEM.

Finally, we tested whether input-specific changes in NMDAR-mediated transmission relate to deficits in motor skills consolidation. We injected mice with ChR2 expressing virus in the motor cortex in control and YAC128 mice and six weeks after the injection we performed single pellet reaching task (Fig. 6*A*); 5 min after the last training session (days 8 and 9, WT = 3.135 ± 0.495, *N* = 2 mice, YAC128 = 0.675 ± 0.67, *N* = 2 mice; Fig. 6*B*), we killed the animals and cut coronal slices. Interestingly, we found that motor training promoted a decrease in AMPA/NMDA ratio at motor cortex to DLS synapses in WT mice (WT naive: 0.632, *n* = 6 neurons, WT after Single pellet: 0.279, *n* = 12 neurons; YAC128 naive: 0.424, *n* = 7 neurons, YAC128 after single pellet: 0.358, *n* = 8 neurons, two-way ANOVA interaction effect *F*_(1,31)_ = 9.282, *p* < 0.05 followed by Bonferroni *post hoc* WT naive vs YAC128 naive, *p* < 0.05; WT naive vs WT after single pellet *p* < 0.001; Fig. 6*B*,*C*). Remarkably we found that motor skills learning-induced synaptic plasticity was absent in YAC128 mice (Fig. 6*B*).

These data not only indicated a circuit-specific form of synaptic plasticity associated with motor skills consolidation but also suggest that this form of plasticity is needed during the consolidation of motor skill learning.

## Discussion

We found that three-month-old YAC128 mice present deficits in consolidation phase of motor skill learning compared with WT without major motor dysfunctions. While during initial task exposition, motor abilities were equivalent between groups, control mice maintained their newly acquired motor skill across training both in rotarod and single pellet reaching tasks whereas YAC128 mice did not. Associated to these alterations, we showed a specific decrease in AMPA/NMDA ratio at motor cortex to DLS synapses in YAC128 mice. Together with a modified NMDAR transmission and enhanced ifenprodil sensitivity in motor cortex to DLS synapses in these mice, we also observed an aberrant NMDAR-dependent LTD induced by optogenetic low-frequency stimulation protocol. Moreover, we highlighted that synaptic plasticity induced by single pellet reaching task training at motor cortex to DLS synapses was occluded in YAC128 mice.

As previously reported (Chiu et al., 2011) and contrary to another report (Slow et al., 2003), we did not observe changes in locomotor activity in YAC128 mice compared with control between 11 and 14 weeks in the circular corridor and in the open field task. Despite the absence of any gross motor impairment, here for the first time we observed a deficit in motor skill consolidation in YAC128 compared with WT. Intriguingly, YAC128 and control mice presented an equivalent motor performance during early phases of motor learning. Remarkably, while WT mice maintained their acquired learning, YAC128 mice showed a worsening of the motor skills after a break in the training session suggesting that YAC128 mice present deficits in the consolidation of motor skill learning. Despite YAC128 mice from C57B6/J background strain which express the htt mutant transgene are not the strain that present the strongest phenotype severity compared with YAC128 mice from a FVB/N background strain (Van Raamsdonk et al., 2007), here we have shown that motor consolidation deficits are detected at early stage in this mouse model of HD. One limitation of this present study is the potential difference in parental behavior that we ca not exclude with our breeding strategy between WT C57B6/J control mice and YAC128 homozygous from C57 B6/J background (for details, see Materials and Methods). Future studies will be needed to investigate whether these deficits are specific within the context of motor learning or if more general deficits in memory consolidation could be observed in these mice.

In this study, we highlighted the importance to distinguish prior initial motor performance from motor skill acquisition and consolidation in order to better detect phenotype abnormality in YAC128 mice. Previous studies have already pinpointed motor dysfunctions in YAC128 mice in the rotarod without defining whether the defects were due to a motor learning impairment or rather a deficit in the consolidation of prior newly acquired motor skill (Van Raamsdonk et al., 2007; Pouladi et al., 2009). Learning new skills is characterized by an initial phase of rapid improvement followed by a more gradual phase of progress as skills are automatized. Interestingly, region-specific changes in neuronal activity and synaptic plasticity in the striatum have been observed during acquisition and consolidation of motor skills (Yin et al., 2009). Using the single pellet reaching task to observe the acquisition and consolidation of new motor skills (Karni et al., 1998), our data support the findings that motor cortex to DLS plasticity during extended training is necessary to the consolidation of motor skills. Importantly, skill reaching task can be considered as a useful motor learning task that can be performed in premanifest HD mouse model where new alternative protective treatments may be tested with a strong potential translational perspective for HD patients (Klein et al., 2011, 2012). Consequently, a detailed analysis of distinct motor tasks seems essential to better dissect motor alterations in initial stage of the disease both in rodents and in humans.

Although we decreased potent stressful environmental effects and considered anxiogenic traits of YAC128 mice by performing handling, habituation and using low light setting conditions, motor tasks cannot be exclusively restricted to motor function. Indeed, deficits in motor task performance could be the consequence of anxiety phenotype, attention and/or motivation deficits. The increase floating behavior during the swimming tank test and decrease time in open arms in the elevated plus maze suggest that premanifest HD mice present anxiety-like and depressive-like phenotypes. Interestingly, previous studies have reported similar results in YAC128 mice and in the R6/2 mice model of HD (Carter et al., 1999; Chiu et al., 2011). Future studies will need to further investigate these behavioral traits in YAC128 mice and examine the relevant circuits. Importantly, we reported the same number of total attempts in single pellet reaching task across days in WT and YAC128 mice, suggesting that both groups attempt to perform this task. Concerning anhedonia evaluation of YAC128 mice, we found different results with sucrose consumption experiment compared with Pouladi et al. (2009). These apparent discrepancies may be first explained by distinct protocols. First, in the Pouladi et al. (2009) study, mice were only exposed one time to the 2% sucrose solution which design cannot exclude the potential confounding effect induced by neophobia without previous food restriction. In our present study, we wanted to evaluate the sucrose preference of mice that underwent a single reaching motor learning under food restriction condition. We therefore exposed during 2 d the animals to 1% of sucrose solution to avoid any confounding effect of the first exposure of sucrose followed by 8% sucrose solution exposure. Under these specific conditions, we did not detect difference in the sucrose consumption. In addition, we used different mice background (in our study: YAC128 from C57 B6/J vs YAC128 from FVB/N background in Pouladi et al., 2009). As reported in Van Raamsdonk et al. (2007), YAC transgene expressing mutant htt is penetrant on both background but the severity is modulated by strain which may also explain these differences. Even if no major anhedonia has been suggested here, the integrity of reward circuits and of dopamine neuromodulation should be further investigated notably by assessing a progressive ratio schedule to control for any general impairment in general motivation in YAC128 mice.

Previous studies pinpointed abnormalities in glutamatergic transmission onto MSN in different HD mouse model across ages (André et al., 2011; Marco et al., 2013). Despite of no significant changes in the PPR at motor cortex to DLS, we noted a trend at 100-ms pulse interval (Fig. 4*L*) in YAC128 mice suggesting that further changes in presynaptic release properties may appear at later stages of the disease. It has been shown that striatal MSNs express higher level of extrasynaptic NMDARs at presymptomatic stages (Okamoto et al., 2009; Milnerwood et al., 2010) and that changes in NMDAR localization are independent of the source of glutamatergic input (Kolodziejczyk and Raymond, 2016). Here, we unraveled specific deficiencies of glutamatergic transmission at motor cortex to DLS synapses whereas no AMPA/NMDA ratio changes were observed at thalamo-striatal synapses or at motor cortex to DMS synapses (data not shown) at that stage neither. In the present study, we used both males and females. Although we did not perform statistical analysis for a specific sex effect, we found important to display males and females data. It will be interesting to further extend this study on sex differences with the progression of HD.

Motor learning deficit in HD mouse models have been linked to deficits in striatal plasticity and to aberrant function of NMDARs. Indeed, deletion of striatal NMDAR abolished striatal long-term potentiation (LTP) and impaired learning (Dang et al., 2006). Furthermore, R6/2 mice show less NMDA-dependent LTP in the striatum compared with WT control (Kung et al., 2007) while deficit in endocannabinoid-dependent LTD was observed in the YAC128 mice (Sepers et al., 2018). Here we show that at motor cortex to DLS synapses, the increased contribution of NMDAR is accompanied by an increase in GluN2B-containing NMDARs. Interestingly, an increase in NMDAR-mediated over an AMPAR-mediated current has been shown in DLS synapses after extended motor training (Yin et al., 2009). Here, we show that synaptic plasticity induced by motor learning does not occur in YAC128 mice, suggesting that the increased number of NMDARs at motor cortex occludes the motor learning-dependent insertion of NMDARs.

Remarkably we also found that NMDA-dependent LTD is aberrant at motor cortex to DLS synapses suggesting that depotentiation of NMDA synaptic transmission in this pathway may restore synaptic transmission and may therefore represent a possible therapeutic intervention.

In general, this study proposed new meaningful insight in the synaptopathic mechanisms of HD. We highlight that deficit in motor skill consolidation-dependent synaptic plasticity at motor cortex to DLS synapses represents an early biomarker for HD. Lastly, we encourage detailed motor investigations at premanifest stage to further screen new potential therapeutically preventive strategies for HD.

## Synthesis

Reviewing Editor: Pablo Castillo, Albert Einstein College of Medicine Decisions are customarily a result of the Reviewing Editor and the peer reviewers coming together and discussing their recommendations until a consensus is reached. When revisions are invited, a fact-based synthesis statement explaining their decision and outlining what is needed to prepare a revision will be listed below. The following reviewer(s) agreed to reveal their identity: NONE. Note: If this manuscript was transferred from JNeurosci and a decision was made to accept the manuscript without peer review, a brief statement to this effect will instead be what is listed below.

Your manuscript has been evaluated by two experts in the field. While both reviewers appreciate the potential significance of your findings, which could provide clues on the pathogenesis of HD during early stages, they also raised a number of concerns that you must address before your manuscript can be considered for publication in eNeuro. Chief among these concerns is the confounding effects of the genetic background, which you should at least acknowledge in a revised manuscript. The editorial decision is based on the reviewers’ specific points (see below), as well as the online discussion that followed. We look forward to receiving a thorough revision of your manuscript.

--------------------------------------------------------------

Reviewer 1

Gangletas et al report early defects in fine motor skill learning and correlated changes in synaptic plasticity in a commonly used model of HD, the YAC128 mouse. Alterations in synaptic transmission and plasticity seem to selectively affect motor cortex to dorsolateral striatum (DLS) synapses. The work is interesting, well-performed and adds to existing literature on early deficits in HD. A selective involvement of the motor-DLS circuit at early stages could provide clues to understand disease initiation and progression. However, the authors do not demonstrate a link between circuit and behavioral deficits as the title seems to suggest, but just correlation.

1) Regarding selectivity and correlation with early behavioral deficits, it would be important to see whether synaptic transmission is unaffected in dorsomedial striatum.

2) Important relevant literature reporting early changes in motor function, motor learning, anhedonia or depressive behavior in YAC128 mice should be cited (Pouladi et al, Brain 2009; van Raamsdonk et al JN 2005). Authors should discuss why their findings differ from the previous work, and comment on differences in YAC128 genetic backgrounds between their study and previous (see van Raamsdonk et al Neurobiology of disease 2007 for phenotypic penetrance across strains).

3) In regard to the floating behavior and the involvement of motivation and emotional rather than motor problems, it would be important to present another measure of depressive behavior apart from anhedonia.

4) Figure 4L and Figure 5E, F seem to point towards a defect in PPR at longer ISIs. Related to this, the numbers provided by the authors in page 16 of the main text are wrong.

5) The enhanced NMDAR-dependent LTD (Figure 5) seems the most striking early phenotype. Is this related to the enhanced GluN2B content shown in Figure 4I, J?

6) The lack of plasticity in AMPA/NMDA ratios in YAC128 mice is intriguing and quite interesting. Is this due to defective NMDAR or AMPAR responses?

7) The writing is confusing, particularly in the behavioral section, and I found many typos. This should be corrected.

Reviewer 2

This manuscript from Glangetas and colleagues has been transferred to eNeuro, after previously being reviewed and revised at The Journal of Neuroscience. During the previous rounds of review, one of the major concerns related to lack of experimental control over the effects of genotype reported in the manuscript, as the genotype effect was confounded with other sources of experimental variation.

First, homozygous YAC128 mutant mice were generated by homozygous YAC128 parents, and compared with wild-type control animals generated by wild-type parents. The breeding strategy means that experimental and control mice may be different due to their own genotype, or due to differences in parental behavior caused by the genotype of dam/sire. This caveat is now stated more explicitly in the Material and Methods section, which is an adequate response, although a more extensive discussion of this limitation would be welcome. In the rebuttal letter, the authors respond to this point by mentioning replication of their effects across cohorts - the concern here is not reproducibility of the effect, but instead the source of variation that drives the effect (genotype of mouse versus parents).

Second, LTD data in Figure 5 comes from wild-type males and YAC128 females. This means that the first two groups of data shown in Figure 5D are different in terms of both genotype and sex, so it is difficult to know whether the difference between groups is caused by genotype or sex. Ideally, the authors would collect a small sample of LTD data from the opposite sex of each genotype, and confirm it is in the same range (since the effect appears very robust). The text added to the Discussion (lines 575-578) addresses the wrong issue: the problem is not that the authors used both male and female mice, which is a strength of the study that I applaud. Instead, the problem is that in this specific data set (Figure 5D), it is difficult to tell whether group differences are caused by genotype or sex.

Third, YAC128 mice were intercrossed with Drd1-tdTomato reporter mice, in order to identify D1-MSNs for whole-cell patch-clamp recordings. In the rebuttal document, the authors break down behavior and physiology data, comparing mice carrying and lacking the Drd1-tdTomato allele. Data in rebuttal Figure 1A are confusing: the legend indicates they are YAC128 mice, but this seems inconsistent with data shown in Figure 1H of the paper, where YAC128 mice take 20+ seconds to cross on D3/D7. Some clarification is needed here.

Based on the analyses shown in the rebuttal, it does not appear that the presence of the reporter allele has a substantial impact on the phenotype of either YAC128 or wild-type mice. However, the manuscript would be improved if these findings were mentioned in the text itself, even as “data not shown”. For example, the sentence spanning lines 139-140 could end as follows: “as similar phenotypes were observed regardless of Drd1-tdTomato genotype (data not shown)”. A rigorous way to address this concern would be to include “Drd1a-tdTomato genotype” as a variable in factorial ANOVA analysis, and report statistical results for main effects and interactions involving this factor as extended data.

One issue that still remains to be clarified is the copy number of the Drd1-tdTomato transgene. The description of the breeding strategy indicates that at least some of the Drd1+ mice were homozygotes for this transgene, while others were likely heterozygous. Was any attempt made to differentiate homozygosity versus heterozygosity for Drd1-tdTomato, in either YAC128 or wild-type mice? If the answer is “no”, this should be stated in the text of the manuscript, and perhaps discussed as another limitation.

## Author Response

Reviewer 1

Gangletas et al report early defects in fine motor skill learning and correlated changes in synaptic plasticity in a commonly used model of HD, the YAC128 mouse. Alterations in synaptic transmission and plasticity seem to selectively affect motor cortex to dorsolateral striatum (DLS) synapses. The work is interesting, well-performed and adds to existing literature on early deficits in HD. A selective involvement of the motor-DLS circuit at early stages could provide clues to understand disease initiation and progression. However, the authors do not demonstrate a link between circuit and behavioral deficits as the title seems to suggest, but just correlation.

1) Regarding selectivity and correlation with early behavioral deficits, it would be important to see whether synaptic transmission is unaffected in dorsomedial striatum.

We thank the reviewer to highlight the crucial point of the alteration specificity which is raised in this study at the motor cortex to dorsolateral striatum synapses in YAC128 mice at early stage.

Indeed, no excitatory synaptic transmission difference was detected with electrical stimulation (Figure 3), neither with optogenetic stimulation of glutamatergic thalamic input (Figure 4 B) but instead we highlight alteration at motor cortex to dorsolateral striatum (Fig 4-6).

Interestingly, it has been shown that the dorsomedial striatum is recruited in the early phase of the acquisition of a motor skill learning task (day 1) whereas the dorsolateral is activated in the late phase (day 8) (Yin et al 2009). Despite of the absence of motor performance difference at day 1 between groups in the rotarod and single pellet reaching task (Figure 1F and Figure 2D), we cannot exclude that the synaptic transmission at a different synapse such as the motor cortex to dorsomedial striatum is unaffected.

Thus, as suggested by the reviewer we now further verify the integrity of the motor cortex to dorsomedial striatum in YAC128 mice. We didn’t see difference at motor cortex to dorsomedial striatum between WT and YAC128 mice (as reported in the figure 1 in the rebuttal letter). These new results confirm and strengthen the selective deficit occurring at motor cortex to dorsolateral striatum in YAC128 mice.

Figure 1. AMPA/NMDA ratio in DMS of male WT and YAC-128 mice after infection in M1 and M2 with ChR2 under a synapsin promoter.

We now report this information in the discussion part of the manuscript:

“Here, we unraveled specific deficiencies of glutamatergic transmission at motor cortex to DLS synapses whereas no AMPA/NMDA ratio changes were observed at thalamo-striatal synapses at that stage neither at motor cortex to DMS synapses (data not shown).”

2) Important relevant literature reporting early changes in motor function, motor learning, anhedonia or depressive behavior in YAC128 mice should be cited (Pouladi et al, Brain 2009; van Raamsdonk et al JN 2005). Authors should discuss why their findings differ from the previous work, and comment on differences in YAC128 genetic backgrounds between their study and previous (see van Raamsdonk et al Neurobiology of disease 2007 for phenotypic penetrance across strains).

We thank the reviewer for this comment and now add in the manuscript these references and further comment them.

(Added in line533) Despite YAC128 mice from C57B6/J background strain which express the htt mutant transgene are not the strain that present the strongest phenotype severity compared to YAC128 mice from a FVB/N background strain (Van Raamsdonk et al 2007), we have shown here that motor consolidation deficits are detected at early stage in this mouse model of Huntington’s disease.

In this study, we highlight the importance to distinguish prior initial motor performance from motor skill acquisition and consolidation to better detect phenotype abnormalities in YAC128 mice. Previous studies have already pinpointed motor dysfunctions in YAC128 mice in the test day in the rotarod following several days of training without defining whether the defect was due to a motor learning impairment or rather a deficit in the consolidation of prior newly acquired motor skill (Pouladi et al 2009, Van Raamsdonk et al 2007).

In addition, here we show alteration in fine motor skill learning consolidation with the single pellet reaching task in YAC128 mice at early stage. This test is already used in clinical trials for Huntington patients (Klein et al 2012) and may further represent an opportunity to test new pharmacological therapeutical strategy in mouse model of Huntington’s disease.

(added line 561)” Concerning anhedonia evaluation of YAC128 mice, we found different results with sucrose consumption experiment compared with Pouladi et al. 2009. These apparent discrepancies may be first explained by distinct protocols. First, in Pouladi et al. study, mice were only exposed one time to the 2 % sucrose solution which design can ‘not exclude the potential confounding effect induced by neophobia without previous food restriction’. In our present study, we wanted to evaluate the sucrose preference of mice that underwent a single reaching motor learning under food restriction condition. We therefore exposed during 2 days the animals to 1 % of sucrose solution to avoid any confounding effect of the first exposure of sucrose followed by a 8% sucrose solution exposure. Under these specific conditions, we didn’t detect difference in the sucrose consumption. In addition, we used different mice background (in our study: YAC128 from C57 B6/J vs YAC128 from FVB/N background in Pouladi et al 2009). As reported in Van Raamsdonk et al 2007, YAC transgene expressing mutant htt is penetrant on both background but the severity is modulated by strain which may also explain these differences.”

3) In regard to the floating behavior and the involvement of motivation and emotional rather than motor problems, it would be important to present another measure of depressive behavior apart from anhedonia.

Effectively, it has been previously suggested that YAC128 mice present floating behaviour in the swimming tank test ((Pouladi et al 2009, Chiu et al 2011). These findings were confirmed here (Figure 1J,K). It remains still controversial whether floating behaviour represents an adaptive strategy to cope with the effort required in the swimming tank test or a depressive state. In addition, it has been previously reported that a combination of anti-depressive treatment (lithium and valproate) notably reduced the immobility time both in the forced swim task and tail suspension task of YAC128 mice which are two classical tests to evaluate depressive like behaviours (Chiu et al 2011).

Despite anxiety trait observed in YAC128 mice, initial motor performances were equivalent between groups in low light conditions. For the single pellet reaching task, YAC128 mice under food restriction performed the same number of total attempts across training compared to WT and therefore develop equivalent sucrose preference. Despite of anxiety and potential depressive states, our experimental conditions allow mice to be motivated to perform this fine skill motor task. However, as we reported in the discussion, even if no major anhedonia has been suggested here, the integrity of reward circuits and of dopamine neuromodulation should be further investigated notably by assessing a progressive ratio schedule to control for any general impairment in general motivation in YAC128 mice.

4) Figure 4L and Figure 5E, F seem to point towards a defect in PPR at longer ISIs. Related to this, the numbers provided by the authors in page 16 of the main text are wrong.

We apologize for this mistake and made the correction.

5) The enhanced NMDAR-dependent LTD (Figure 5) seems the most striking early phenotype. Is this related to the enhanced GluN2B content shown in Figure 4I, J?

We appreciate this interesting point raised by the reviewer. We found an enhanced LTD at motor cortex to dorsolateral striatum synapses with low frequency stimulation protocol in YAC128 mice (Fig 5B) blocked by NMDAR antagonist application APV (Fig 5C,D). In addition, we observed an enhanced GluN2B content specifically at that synapses in YAC128 mice (Fig 4 I, J) which may confer greater calcium permeability properties (Paoletti et al 2013) and therefore may contribute to enhanced aberrant NMDAR dependent LTD.

6) The lack of plasticity in AMPA/NMDA ratios in YAC128 mice is intriguing and quite interesting. Is this due to defective NMDAR or AMPAR responses?

Previous works have already shown enhance expression of NMDA receptor at MSN synapses (Levine et al 1999, Milnerwood et al 2010). We further show enhanced expression of GluN2B specifically at motor cortex to DLS synapses in YAC128 mice (Figure 4 I, J). However, we didn’t see changes in the amplitude of the optically-induced AMPAR mediated current (Figure 4 F) as well as in the amplitude of strontium evoked asynchronous AMPAR events (Figure 4G), neither in the rectification index of AMPA- mediated transmission (Figure 4 E). Altogether, these data suggest that a defect in NMDAR-mediated transmission might be responsible for the absence of synaptic plasticity after motor skill reaching learning in YAC128 mice. As we proposed in the discussion, a depotentiation of NMDA synaptic transmission in this pathway may restore synaptic transmission and may crucial to rescue motor skill consolidation in motor skill reaching task.

7) The writing is confusing, particularly in the behavioral section, and I found many typos. This should be corrected.

We apologize for typos and we corrected them.

Reviewer 2

This manuscript from Glangetas and colleagues has been transferred to eNeuro, after previously being reviewed and revised at The Journal of Neuroscience. During the previous rounds of review, one of the major concerns related to lack of experimental control over the effects of genotype reported in the manuscript, as the genotype effect was confounded with other sources of experimental variation.

First, homozygous YAC128 mutant mice were generated by homozygous YAC128 parents, and compared with wild-type control animals generated by wild-type parents. The breeding strategy means that experimental and control mice may be different due to their own genotype, or due to differences in parental behavior caused by the genotype of dam/sire. This caveat is now stated more explicitly in the Material and Methods section, which is an adequate response, although a more extensive discussion of this limitation would be welcome. In the rebuttal letter, the authors respond to this point by mentioning replication of their effects across cohorts - the concern here is not reproducibility of the effect, but instead the source of variation that drives the effect (genotype of mouse versus parents).

We thank the reviewer for this comment and we have now completed the discussion session considering this limitation.

“One limitation of this present study is the potential difference in parental behavior that we can’t exclude with our breeding strategy between wild-type C57B6/J control mice and YAC128 homozygous from C57 B6/J background (see details in the materiel and method section).”

Second, LTD data in Figure 5 comes from wild-type males and YAC128 females. This means that the first two groups of data shown in Figure 5D are different in terms of both genotype and sex, so it is difficult to know whether the difference between groups is caused by genotype or sex. Ideally, the authors would collect a small sample of LTD data from the opposite sex of each genotype, and confirm it is in the same range (since the effect appears very robust). The text added to the Discussion (lines 575-578) addresses the wrong issue: the problem is not that the authors used both male and female mice, which is a strength of the study that I applaud. Instead, the problem is that in this specific data set (Figure 5D), it is difficult to tell whether group differences are caused by genotype or sex.

We thank the reviewer for this point. In the revision, we have now performed new experiments and we have added 5 neurons from WT females and 5 neurons from YAC128 males (here displayed in Figure 2. New data, Grey wild type and blue YAC128). As expected, the effect we have previously observed is not sex dependent. We merged these new data with the previous one and the data are now reported in the Fig. 5B and D

Figure 2.

Third, YAC128 mice were intercrossed with Drd1-tdTomato reporter mice, in order to identify D1-MSNs for whole-cell patch-clamp recordings. In the rebuttal document, the authors break down behavior and physiology data, comparing mice carrying and lacking the Drd1-tdTomato allele. Data in rebuttal Figure 1A are confusing: the legend indicates they are YAC128 mice, but this seems inconsistent with data shown in Figure 1H of the paper, where YAC128 mice take 20+ seconds to cross on D3/D7. Some clarification is needed here.

We thank the reviewer for pointing this. The data showed in the rebuttal document we have previously submitted, correspond to analysis of the swimming tank test suggested by the previous reviewer. In that analysis, we had excluded the failed trials (threshold 30 sec). This was done to show that there were no differences between genotypes. The figure in the manuscript (Fig 1 H) includes the failed trails at the maximum threshold time (30 sec). We apologize for this confusion.

Based on the analyses shown in the rebuttal, it does not appear that the presence of the reporter allele has a substantial impact on the phenotype of either YAC128 or wild-type mice. However, the manuscript would be improved if these findings were mentioned in the text itself, even as “data not shown”. For example, the sentence spanning lines 139-140 could end as follows: “as similar phenotypes were observed regardless of Drd1-tdTomato genotype (data not shown)”. A rigorous way to address this concern would be to include “Drd1a-tdTomato genotype” as a variable in factorial ANOVA analysis, and report statistical results for main effects and interactions involving this factor as extended data.

We appreciate the reviewer advice and now add this sentence “as similar phenotypes were observed regardless of Drd1-tdTomato genotype (data not shown)”.

One issue that still remains to be clarified is the copy number of the Drd1-tdTomato transgene. The description of the breeding strategy indicates that at least some of the Drd1+ mice were homozygotes for this transgene, while others were likely heterozygous. Was any attempt made to differentiate homozygosity versus heterozygosity for Drd1-tdTomato, in either YAC128 or wild-type mice? If the answer is “no”, this should be stated in the text of the manuscript, and perhaps discussed as another limitation.

We correct the manuscript and now specify that we didn’t differentiate the homozygosity versus heterozygosity for DrD1-tdTomato which can be a limitation in this study in the material and method section.
